# Association of Alcohol Consumption with Markers of Prostate Health and Reproductive Hormone Profiles: A Multi-Center Study of 4535 Men in China

**DOI:** 10.1371/journal.pone.0142780

**Published:** 2015-11-11

**Authors:** Meng Rao, Lian-Dong Zuo, Fang Fang, Kuete Martin, Yi Zheng, Hui-Ping Zhang, Hong-Gang Li, Chang-Hong Zhu, Cheng-Liang Xiong, Huang-Tao Guan

**Affiliations:** 1 Family Planning Research Institute, Tongji Medical College, Huazhong University of Science and Technology, Wuhan, China; 2 Guangzhou Institute of Population and Family Planning, Guangzhou, China; 3 Department of Venereology, Wuhan Institute of Dermatology and Venereology, Wuhan, China; 4 Reproductive Medicine Center, Tongji Medical College, Huazhong University of Science and Technology, Wuhan, China; University of Sydney, AUSTRALIA

## Abstract

**Background:**

The effect of alcohol consumption on prostate health and reproductive hormone profiles has long been investigated and currently, no consensus has been reached. Additionally, large studies focusing on this topic are relatively rare in China.

**Purpose:**

To investigate the association of alcohol consumption with prostate measurements and reproductive hormone profiles in Chinese population; and to examine the relationship between hormone levels and prostate measurements.

**Methods:**

This cross-sectional study included 4535 men from four representative provinces of China. Demographic details, family history of prostate disease, tobacco and alcohol consumption, as well as International Prostate Symptom Score (I-PSS) were collected through a questionnaire. Total prostate specific antingen (total PSA), free PSA, free PSA/total PSA ratio (f/tPSA), and reproductive hormones were measured in serum. Multi-variable regression models were used to test for association of alcohol consumption with markers of prostate health, used to test for association of alcohol consumption with reproductive hormones, and reproductive hormones with markers of prostate health.

**Results:**

Alcohol consumption had no obvious impact on total PSA concentration and I-PSS. Current drinkers had lower level of free PSA (β = -0.11, *p* = 0.02) and f/tPSA (β = -0.03, *p* = 0.005), former drinkers also had lower level of free PSA (β = -0.19, *p* = 0.02) when compared with never drinkers. Lower Luteinizing hormone (LH) (β = -1.05, *p* = 0.01), sex hormone-binding globulin (SHBG) (β = -4.71, *p* = 0.01) and higher estradiol (β = 7.81, *p* = 0.01) was found in current drinkers than never drinkers, whereas higher LH (β = 1.04, *p* = 0.04) and free testosterone (FT) (β = 0.03, *p* = 0.02) was detected in former drinkers than never drinkers. Furthermore, LH was positively associated with f/tPSA (β = 0.002, *p* = 0.006), SHBG was also positively related with free PSA (β = 0.003, *p* = 0.003) and f/tPSA (β = 0.0004, *p* = 0.01). Both total testosterone (TT) and FT were inversely related with I-PSS (OR = 0.97, 95% CI, 0.95–0.98; OR = 0.23, 95% CI, 0.11–0.45, respectively).

**Conclusions:**

Alcohol consumption could affect serum free PSA concentration and also f/tPSA ratio, and also acts as an endocrine disruptor on the male reproductive hormone profiles. LH and SHBG were positively related with fPSA and f/tPSA, and higher level of TT and FT may be helpful for improving participants' subjective symptoms.

## Introduction

As a very common lifestyle exposure, alcohol consumption has been investigated in several studies to identify whether it played a role in the occurrence of prostate diseases, such as prostatitis, benign prostatic hyperplasia (BPH) and prostate cancer (PCa). However, consistent conclusions have not been reached to date. Some of those studies showed that alcohol was a risk factor for prostatitis [1,2] and a protective factor for BPH [3,4]. Long-term alcohol consumption may be etiologically relevant to PCa [5]. However, other investigators draw different conclusions [6,7].

As the most valuable marker for screening and early diagnosis for prostate tumor, prostate-specific antigen (PSA) exists as either complexed or free form in blood. Complexed PSA primarily bound to α-1-antichymotrypsin and α-2-macroglobulin. The diagnostic value of total PSA is limited by its lack of specificity, because total PSA levels may also be raised in BPH and prostatitis. The proportion of free PSA (f/tPSA) in the serum is higher in BPH than in PCa, and measurement of the f/tPSA has therefore been recommended to improve diagnostic accuracy when total PSA levels were between 4–10 ng/ml [8]. Few investigations have focused on the effect of alcohol consumption on these markers. Jorge’s study showed that alcohol consumption could significantly elevate serum total PSA levels in prostate tumor patients [9]. Experimental research carried out in rats also indicated that alcohol could increase serum total PSA [10]. The International Prostate Symptom Score (I-PSS) is a widely used method to assess symptom of prostate diseases in patients, and is also usually used to assess the presence and degree of lower urinary tract symptoms (LUTS) [11,12]. Limited studies have investigated the association of alcohol consumption with LUTS, some reported a direct association [13,14] and others draw the opposite conclusions [15,16]. A population-based study in South Korea reported that alcohol consumption presented a J-shaped association with lower urinary tract symptoms, namely moderate alcohol drinkers (0–10 g/day) have a lower value of I-PSS than never drinkers, whereas heavy drinkers (>40 g/day) have a higher value of I-PSS than never drinkers [12]. Alcohol intake varies in age, region, culture and education [17]; and can be influenced by dietary habit and other health behaviors [18]. Inconsistency in research findings regarding the effect of alcohol on prostate measurements may reflect different patterns of covariation with important, but unidentified confounders.

Reproductive hormones, especially androgens have been shown to play a critical role in the prostate development and maintenance, particularly in the prostatic epithelium [19]. Investigators have suggested that alcohol intake could play a role in the etiology of both BPH and PCa through its impact on endocrine factors, because alcohol drinking, especially heavy drinking could influence androgen metabolism and disrupt the reproductive hormone balance [20,21]. As a consequence, we were interested in the impact of alcohol consumption on reproductive hormone profiles and also the association of hormone levels and prostate measurements.

Currently, large studies focused on the effect of alcohol on prostate measurements are relatively rare, especially in China. Therefore, we carried out this study among Chinese population to test for an association between alcohol consumption and prostate serum markers (total PSA, free PSA, f/tPSA), symptoms (I-PSS) as well as reproductive hormone profiles; and to examine the relationship between reproductive hormone levels and prostate measurements.

## Participants and Methods

### Ethics statement

The project proposal was approved by the Ethics Committee of Tongji Medical College, Huazhong University of Science and Technology (No. 20130311), and conducted in accordance with its guidelines. Written informed consent was obtained from every participant enrolled in this study.

### Study design

This cross-sectional multicenter study was carried out between March 2013 and May 2014. To make a comprehensive investigation in a residential, geographic and age representative sample of Chinese men, our research institute collaborated with four provincial health and family planning commission which are geographically and demographically representative of the country: Jiangsu (east coast), Guangdong (southern China), Hubei (central China) and Guizhou (south-west China). These four provinces vary in their level of economic development, from southeastern coast to central area, and then to western China. From each province, approximately 1000–1300 participants were enrolled in the present study.

We used a two-stage sampling strategy to recruit participants. Firstly, we selected 4 districts from each of the participating provinces, the selected districts should be representative of the province according to the characteristics of geography, economy and also population density. A total of 16 districts with mixed urban and rural locations from the four participating provinces were selected finally for this study. Secondly, we recruited volunteers from the 16 participating districts. According to the protocol, staffs from local health and family planning commission distributed posters to each family. The staffs expounded the design and purpose of this study to all potential subject and encouraged them to go to the the family planning clinics to receive a free medical examination. The subjects who came to the family planning clinics were recruited if they met the eligibility criteria of the study.

### Study population

The inclusion criteria in this study were as follows: men 20 years old or older; normally developed; could communicate with the investigators and complete the questionnaires. The exclusion criteria were as follows: they had severe brain, cardiovascular or kidney diseases; they suffered from reproductive endocrine disorders; they used steroids in the past 3 months; they reported current prostate infection or inflammation, rectal examination in the past week, or a prostate biopsy or cystoscopy in the past month. A total of 4579 subjects were recruited and screened into the study. However, 44 were excluded from the analysis due to unclear recording of alcohol consumption. Thus, 4535 subjects were eligible and enrolled into the data analysis. Of these, 1121 came from Guangdong, 1056 from Hubei, 1141 from Jiangsu and 1217 from Guizhou province. All subjects were asked to complete a questionnaire and blood collection.

### Questionnaires

Participants completed a questionnaire in a face-to-face interview. The questionnaire included detailed questions about demographic and anthropometric characteristics (age, height, weight and race), family history of prostate disease, tobacco and alcohol consumption. Body mass index (BMI) was calculated from weight (in kg) divided by height (in metres) squared. Tobacco use was categorized into three groups of never (if they have smoked less than 100 cigarettes in their entire life), current (if they have smoked more than 100 cigarettes and smoked in the previous year) and former (if they have quitted smoking in the previous year). Alcohol consumption status was categorized into three groups of never drinking (if participants have consumed less than 12 drinks in their entire life), current drinking (if participants have consumed more than 12 drinks and drunk during the past year) and former drinking (if participants have quitted drinking in the past year). Estimate of usual weekly alcohol intake of current drinkers was calculated by combining intake amount and frequency separately for beer and wine, approximately, 12 bottles of beer can be converted to 0.5 kg of alcohol. Current drinkers were categorized into three groups according to the alcohol consumption amount in the previous year: <0.25 kg/week, 0.25–1 kg/week and >1 kg/week. Furthermore, the current drinkers were also divided into two groups according to the reported duration of alcohol intake: ≤15 years and >15 years.

I-PSS was collected from a 7-item questionnaire including incomplete emptying, frequency, intermittency, urgency, weak stream, hesitancy, and nocturia. A patient gives each symptom a rating from 0 to 5 based on its severity over the past month. These are summed to produce an overall severity score, which is typically used to categorize patients as having none to mild (score 0–7), moderate (8–19), or severe (20–35) symptoms [12].

### Blood sampling and PSA/ hormone assays

Venous blood specimen was acquired from each overnight-fasting participant between 7:00–10:00 in the morning after completion of the questionnaire. Blood samples (approximately 8 ml from each participant) were collected into 10-ml vacuum tubes without anticoagulant. After cooling at room temperature, the tubes were centrifuged at 3 000 × *g* for 10 min, and then serum was separated into multiple labeled (ID number only) aliquots and temporarily stored at -80°C, in the testing centers of each local Family Planning clinic. Then the samples stored at each center were preserved in dry ice and transported to the reproductive center of Tongji medical college for analysis. The serum total PSA and free PSA were measured using a chemiluminescent immunoassay method according to the manufacturers’ instructions, on the automated UniCel DxI 800 analyzer (Beckman Coulter, Brea, USA). Briefly, this method employs paramagnetic particles for separation of free and bound analyte fractions and a chemiluminescent substrate for light signal generation. The amount of light produced is proportional to the concentration of the analyte being measured. From this data, f/tPSA was also calculated. Plasma concentrations of follicle-stimulating hormone (FSH), luteinizing hormone (LH), estradiol, total testosterone (TT) and sex hormone-binding globulin (SHBG) were also tested using a chemiluminescent immunoassay method on UniCel DxI 800 analyzer (Beckman Coulter, Brea, USA). The commercial kits were also provided by Beckman Coulter, Inc. Free testosterone (FT) concentration was calculated by the FTZ formula [22]. All of the assays were carried out by the same specialized technician, to minimize the effect of between-assay variability.

### Statistical analysis

Descriptions of alcohol status were compared across categorical participant characteristics using Chi-square tests. The continuous measures total PSA, free PSA, f/tPSA and I-PSS in each group were expressed as Mean ± SD and compared across different alcohol consumption status groups, amount groups, and duration using analysis of one-way ANOVA or a t test, as appropriate. Distributions of participants among different groups were compared using Chi-square test. Multi-variable logistic regression and multiple linear regression models were used to analyze the relationship between alcohol consumption levels and total PSA, free PSA, f/tPSA and I-PSS, the relationship between alcohol consumption and reproductive hormone levels, and also the association of reproductive hormone levels and those prostate measurements. All multivariate models adjusted for age, BMI, race, family history of prostate disease, research center and tobacco consumption habits. All statistical tests were two-sided and p≤ 0.05 was considered statistically significant. Data analysis was conducted using SPSS 17.0 (SPSS Inc., Chicago, IL, USA)

## Results

### Participant characteristics

A total of 4535 subjects (average age 54.7±11.0) are included in the present analysis. Table 1 shows the distributions of categories of alcohol consumption by participant characteristics. Of these subjects, 1260 (27.8%), 2982 (65.8%) and 293 (6.4%) subjects were categorized as never, current and former drinkers, respectively. Of the 2982 current alcohol drinkers, 821 (27.5%) drank more than 1 kg/week, and 2151 (72.1%) drank more than 15 years (Table 1).

**Table 1:** Demographic and clinical characteristics of participants, expressed as percent distributions, according to level of alcohol consumption.

Characteristics	Alcohol consumption status				Alcohol consumption amount (kg/week)				Alcohol consumption duration (years)		
	Never	Current	Former	P-value	<0.25	0.25–1	>1	P-value	≤15	>15	P-value
	(n = 1260)	(n = 2982)	(n = 293)		(n = 1297)	(n = 864)	(n = 821)		(n = 831)	(n = 2151)	
**Age, years (n = 4535)**											
**<30**	0.64	0.72	0		1.02	0.35	0.62		2.22	0.09	
**30–40**	6.99	6.94	1.03		10	5.79	3.08		16.93	2.88	
**40–50**	23.23	30.88	15.41		33.46	30.38	24.26		42.89	25.18	
**50–60**	25.56	31.94	25.68		28.19	32.27	36.58		21.51	35.19	
**>60**	43.57	29.51	57.88	0.81	27.32	31.21	35.47	<0.01**	16.44	36.66	<0.01**
**BMI (n = 4476)** ^**a**^											
**<18.5**	6.54	3.98	4.79		3.72	4.2	3.42		3.15	4.21	
**18.5–25**	60.89	56.34	61.64		57.1	53.61	58.85		58.35	55.84	
**>25**	32.56	39.68	33.56	<0.01**	39.18	42.19	37.73	0.86	38.5	39.95	0.86
**Race (n = 4529)** ^**b**^											
**Han**	99.84	99.76	100		99.77	99.88	99.76		99.76	99.77	
**others**	0.16	0.24	0	0.64	0.23	0.12	0.24	0.98	0.24	0.23	0.97
**Family history of prostate disease (n = 4535)**											
**Yes**	7.33	11.46	9.42		13.17	12.06	8.57		15.38	10.48	
**No**	92.67	88.54	90.58	0.03*	86.83	87.94	91.43	0.06	84.62	89.52	0.03*
**Research centre (n = 4535)**											
**Guangdong**	24.1	25.72	17.47		16.14	15.27	42.86		27.24	21.26	
**Hubei**	21.71	24.11	21.58		23.35	30.65	19.41		20.82	25.84	
**Jiangsu**	21.87	26.84	20.89		28.78	32.75	18.68		22.88	28.22	
**Guizhou**	32.32	23.33	40.07	<0.01**	31.73	21.33	19.05	<0.01**	29.06	24.67	<0.01**
**Smoking status (n = 4535)**											
**Never**	41.02	27.98	13.01		31.19	25.41	18.8		28.931	23.83	
**Current**	50.92	61.94	61.62		59.5	62.94	68.99		62.23	64.16	
**Former**	8.06	10.08	26.37	<0.01**	9.31	11.66	12.21	<0.01**	8.84	12.01	<0.01**

BMI, body mass index.

^a^ 59 participants were excluded due to less detailed record of height or weight.

^b^ 6 participants were excluded due to less detailed record of race.

*p< 0.05

**p<0.01

### Prostate measurement results in different alcohol consumption groups

We found that free PSA and f/tPSA varied significantly among different alcohol consumption status groups (*p* = 0.03 and <0.01, respectively), whereas total PSA was not obviously affected. Alcohol consumption amount significantly affected the levels of f/tPSA and I-PSS (*p* = 0.05 and <0.01, respectively), and only I-PSS changed significantly among different alcohol consumption duration groups (*p* = 0.03) (Table 2).

**Table 2:** Prostate measurement results in different alcohol consumption groups [Mean (SD)].

Prostate measurements	Total PSA (ng/ml), n = 2146	Free PSA (ng/ml), n = 2146	f/tPSA, n = 2146	I-PSS, n = 3482
**Alcohol consumption status**				
**Never**	1.49 (2.86)	0.36 (0.67)	0.30 (0.12)	3.48 (5.92)
**Current**	1.38 (3.09)	0.30 (0.41)	0.27 (0.12)	3.20 (5.25)
**Former**	1.30 (1.87)	0.32 (0.35)	0.29 (0.11)	4.32 (6.20)
**P-value**	0.69	0.03*	< 0.01**	0.24
**Alcohol consumption amount**				
**<0.25 kg/week**	1.46 (4.27)	0.31 (0.58)	0.27(0.12)	3.50 (5.35)
**0.25–1 kg/week**	1.31 (2.02)	0.28 (0.29)	0.26(0.11)	3.91 (5.93)
**>1 kg/week**	1.38 (2.49)	0.30 (0.31)	0.28 (0.12)	2.44 (4.58)
**P-value**	0.77	0.54	0.05*	< 0.01**
**Alcohol consumption duration**				
**≤15 years**	1.30 (1.97)	0.30 (0.31)	0.28 (0.12)	2.84 (4.76)
**>15 years**	1.38 (3.48)	0.30 (0.45)	0.27(0.12)	3.52 (5.60)
**P-value**	0.62	0.84	0.38	0.03*

2146 participants from Hubei and Guangdong province received the test of total PSA, free PSA and f/tPSA. 3482 participants from the 4 provinces completed the questionnaire of I-PSS. Total PSA, free PSA, f/tPSA and I-PSS in different alcohol consumption groups were compared using analysis of one-way ANOVA or a t test. PSA, prostate specific antigen. f/tPSA, free PSA/total PSA ratio. I-PSS, In ternational Prostate Symptom Score. SD, standard deviation.

*p< 0.05

**p<0.01

### Reproductive hormone levels in different alcohol consumption groups

The results showed that all six reproductive hormone levels were significantly different among different alcohol consumption status groups (all *p*<0.01). LH and SHBG also varied obviously among different alcohol consumption amount and duration groups (all *p*<0.01). In addition, alcohol consumption duration obviously affected FT levels (*p*<0.01). (Table 3)

**Table 3:** Reproductive hormone levels in different alcohol consumption groups [Mean (SD)].

Prostate measurements	FSH (IU/L)	LH (IU/L)	Estradiol (pg/ml)	TT (nmol/l)	FT (nmol/l)	SHBG (nmol/l)
**Alcohol consumption status**						
**Never**	13.51 (14.50)	7.32 (6.33)	27.34(13.57)	16.76(6.28)	0.23 (0.09)	51.93(26.47)
**Current**	9.87 (5.84)	6.19 (4.23)	31.86 (16.51)	16.41(6.71)	0.24 (0.19)	45.11(23.44)
**Former**	14.52 (12.22)	9.03 (11.28)	28.38 (12.06)	18.03(10.53)	0.28 (0.33)	51.44(28.38)
**P-value**	< 0.01**	< 0.01**	<0.01**	<0.01**	< 0.01**	< 0.01**
**Alcohol consumption amount**						
**<0.25 kg/week**	9.80(6.10)	6.12 (4.76)	32.18(16.48)	16.51(6.54)	0.24 (0.16)	44.52(22.63)
**0.25–1 kg/week**	10.44 (7.18)	5.60 (3.84)	31.44(17.23)	16.04(6.03)	0.24 (0.14)	43.78(22.12)
**>1kg kg/week**	10.96 (7.46)	6.89 (6.08)	30.76 (13.86)	16.81(7.87)	0.25 (0.24)	48.03(25.36)
**P-value**	0.37	<0.01**	0.78	0.07	0.18	<0.01**
**Alcohol consumption duration**						
**≤15 years**	1.30 (1.97)	5.50 (3.29)	28.87 (13.06)	16.57(6.33)	0.25 (0.17)	41.40(21.85)
**>15 years**	1.38 (3.48)	6.59 (5.40)	31.95(15.70)	16.25(6.26)	0.23 (0.16)	47.20(24.21)
**P-value**	0.17	<0.01**	0.07	0.22	< 0.01**	<0.01**

4372 participants from the 4 provinces received reproductive hormone test. Multiple linear regression model was used, adjusted for age, BMI, race, family history of prostate disease, smoking status and research center. TT: total testosterone; FT: free testosterone; SHBG: sex hormone-binding globulin. Ref, reference.

**p< 0.01

### Relationship between alcohol consumption and prostate measurements

After adjustment for the potential confounders, we found that current and former drinkers had significantly lower value of free PSA concentration (β = -0.11, *p* = 0.02 and β = -0.19, *p* = 0.02, respectively), current drinkers also had lower level of f/tPSA ratio (β = -0.03, *p* = 0.005) when taking never drinkers as the reference (Table 4).

**Table 4:** Multi-variable regression model to analyze the relationship between alcohol consumption levels and prostate measurements.

Prostate measurements	tPSA^a^, [β (P-value)]	fPSA^a^, [β (P-value)]	f/tPSA^a^, [β (P-value)]	I-PSS^b^, [OR (95% CI)]
**Alcohol consumption status**				
**Never**	Ref.	Ref.	Ref.	Ref.
**Current**	-0.22 (0.29)	-0.11 (0.02)*	-0.03 (0.005)**	0.87 (0.64–1.18)
**Former**	-0.57 (0.10)	-0.19 (0.02)*	-0.03 (0.84)	0.88 (0.53–1.47)
**Alcohol consumption amount**	0.02 (0.82)	0.001 (0.94)	0.003 (0.64)	1.21 (0.98–1.49)
**Alcohol consumption duration**	0.01 (0.90)	-0.005 (0.50)	-0.006 (0.34)	1.00 (0.81–1.23)

^a^ Multiple linear regression model was used, adjusted for age, BMI, race, family history of prostate disease, dietary habit and smoking status.

^b^ Multi-variable logistic regression model was used, adjusted for age, BMI, race, family history of prostate disease, smoking status and research center.

PSA, prostate specific antigen. f/tPSA, free PSA/total PSA ratio. I-PSS, International Prostate Symptom Score. OR, odds ratio. CI, confidence interval. Ref, reference.

*p<0.05

** *p*<0.01

### Relationship between alcohol consumption and reproductive hormone levels

FSH levels didn’t vary significantly among different alcohol consumption groups. LH in current drinkers was lower than never drinkers (β = -1.05, *p* = 0.01), whereas the value in former drinkers was higher than never drinkers (β = 1.04, *p* = 0.04). As for estradiol, current drinkers had obviously higher level than never drinkers (β = 7.81, *p* = 0.01). Alcohol consumption seemed to have no effect on TT, however, FT in former drinkers was lower when taking never drinkers as the reference. Additionally, current drinkers had obviously lower level of SHBG than never drinkers (β = -4.71, *p* = 0.01), alcohol consumption intensity was negatively associated with SHBG levels (β = -1.58, *p* = 0.03). (Table 5).

**Table 5:** Multi-variable regression model to analyze the relationship between alcohol consumption levels and reproductive hormone levels [β (P-value)].

Sexual hormones	FSH	LH	Estradiol	TT	FT	SHBG
**Alcohol consumption status**						
**Never**	Ref.	Ref.	Ref.	Ref.	Ref.	Ref.
**Current**	-1.57 (0.27)	-1.05 (0.01)**	7.81(0.01)**	-0.49 (0.18)	0.005(0.49)	-4.71 (0.01)**
**Former**	2.26 (0.31)	1.04 (0.04)*	0.77(0.81)	0.58 (0.32)	0.03(0.02)*	-0.95 (0.61)
**Alcohol consumption amount**	0.58 (0.38)	0.11 (0.53)	-0.55(0.72)	0.02 (0.92)	0.01(0.09)	-1.58 (0.03)*
**Alcohol consumption duration**	-1.00 (0.17)	-0.06 (0.79)	0.94(0.52)	-0.07 (0.75)	-0.002(0.62)	0.56 (0.44)

4372 participants from the 4 provinces received reproductive hormone test. Multiple linear regression model was used, adjusted for age, BMI, race, family history of prostate disease, smoking status and research center. TT: total testosterone; FT: free testosterone; SHBG: sex hormone-binding globulin. Ref, reference.

*p< 0.05.

**p< 0.01.

### Relationship between reproductive hormone levels and prostate measurements

SHBG level was positively associated with free PSA (β = 0.003, *p* = 0.003) and also f/tPSA (β = 0.0004, *p* = 0.01). LH was also positively associated with f/tPSA (β = 0.002, *p* = 0.006). Additionally, both TT and FT were negatively associated with I-PSS scores (OR = 0.97, 95% CI, 0.95–0.98 and OR = 0.23, 95% CI, 0.11–0.45, respectively). (Table 6).

**Table 6:** Multi-variable regression model to analyze the relationship between sexual hormones and prostate measurements.

Prostate measurements	PSA^a^ [β (P-value)]	fPSA^a^ [β (P-value)]	f/tPSA^a^ [β (P-value)]	I-PSS^b^ [OR (95% CI)]
**FSH (for one unit increase)**	0.005(0.28)	0.0004(0.64)	-0.001(0.31)	0.99(0.97,1.02)
**LH (for one unit increase)**	-0.007 (0.59)	0.004 (0.28)	0.002 (0.006)**	0.99(0.97,1.02)
**Estradiol (for one unit increase)**	0.005(0.15)	-0.0001 (0.82)	-0.001(0.09)	1.00(0.98,1.01)
**TT (for one unit increase)**	-0.02 (0.12)	-0.001 (0.55)	0.0002 (0.71)	0.97 (0.95,0.98)*
**FT (for one unit increase)**	-0.51(0.25)	-0.09(0.36)	-0.007(0.73)	0.23(0.11, 0.45)*
**SHBG (for one unit increase)**	0.001 (0.73)	0.003 (0.003)**	0.0004 (0.01)*	1.00 (0.99,1.01)

^a^ Multiple linear regression model was used, adjusted for age, BMI, race, family history of prostate disease, dietary habit and smoking status.

^b^ Multi-variable logistic regression model was used, adjusted for age, BMI, race, family history of prostate disease, smoking status and research center.

PSA, prostate specific antigen. f/tPSA, free PSA/total PSA ratio. I-PSS, International Prostate Symptom Score. TT: total testosterone; FT: free testosterone; SHBG: sex hormone-binding globulin. OR, odds ratio. CI, confidence interval.

*p<0.05

** *p*<0.01

## Discussion

Chronic alcohol consumption is regarded as a risk factor for diseases of several systems. But the impact on prostate diseases and also reproductive hormone profiles was largely unclear. This was one of the largest population-based studies in China to investigate the associations of alcohol consumption and prostate measurements as well as reproductive hormone profiles.

There was a clinical and also an animal-based study showing that alcohol consumption could elevate serum total PSA [9,10]. However, another large-population based study in UK found evidence of lower total PSA levels for increasing alcohol consumption [23]. In this study we didn’t find any association between alcohol consumption (status, amount as well as duration) and total PSA levels. Interestingly, we found an obvious lower value of free PSA (*p* = 0.02) and f/tPSA ratio (*p* = 0.008) in current drinkers than never drinkers, and also a significant lower value of free PSA (*p* = 0.02) in former drinkers than never drinkers. However, these variations were not related to alcohol consumption amount and duration (Table 3). Several studies investigated the risk of alcohol consumption on PCa, but currently no consensus has been reached. Most cohort and case-control studies reported no evidence of an association between alcohol consumption and PCa [6,7]. Others reported an effect of alcohol specific to heavy alcohol consumers [24,25], increased frequency of alcohol consumption [26] as well as lifetime alcohol consumption [27,28], although these studies didn’t provide specific PSA values. It has been found that total PSA and its derivatives (free PSA, f/tPSA) were largely related to demographic, ethnic and clinical factors [29,30]. Furthermore, alcohol consumption habit varies in age, region, culture and education [17]; and can be influenced by dietary habit and other health behaviors [18]. Therefore, the inconsistent conclusions may be due to different study populations and sample size. Because of higher level in BPH, the proportion of free PSA in the serum is useful to distinguish the PCa from BPH when total PSA levels were between 4–10 ng/ml [8]. Previous studies indicated that alcohol consumption was associated with a decreased risk of BPH [3,4]. James et al also demonstrated that elevated free PSA levels predict clinical BPH independent of total PSA levels [31]. Therefore, in this study, it is reasonable for us to speculate that the lower values of free PSA and f/tPSA in current and former drinkers may be due to lower incidence of BPH when compared with never drinkers. Our study also found no obvious relationship between alcohol consumption and I-PSS, this is consistent with another study which was also based on a large number of Chinese population [11]. However, other studies draw different conclusions. Joseph *et al* investigated the risk factors for LUTS in a population-based sample of African-American men and found a positive association between heavy alcohol consumption and LUTS in African-American men [13]. Rohrmann *et al*. found that moderate alcohol consumption was protective against LUTS [16]. Different study population and alcohol measurement may also account for the inconsistent conclusions.

Acute and repeated alcohol consumption has been reported to result in transient serum testosterone level decreasing, due to decreased production and increased metabolism, as well as possible increased circulating estrogen concentrations [13,32]. Our present study also found an obviously higher level of estrogen in current drinkers.

However, in this study, no total testosterone or free testosterone variation was observed among different alcohol drinking groups except that free testosterone in former drinkers was higher than never drinkers (*p* = 0.02). Usually drinkers who quit alcohol may be due to some disorders such as cardiovascular disease etc. Thus the variation of free testosterone in former drinkers may be caused by some other disorders which could disrupt the function of gonadal axis. LH level was found lower in current drinkers and higher in former drinkers when taking never drinkers as reference, regardless of age, BMI, tobacco consumption and research centers. LH in men was secreted from hypophysis and primarily promote the synthesis of androgens in Leydig cells [33], the variation of LH levels indicated a functional disruption of alcohol on hypothalamic-pituitary-gonadal axis. Furthermore, we detected a lower level of SHBG in current drinkers, and the value was negatively associated with alcohol consumption amount (Table 4). SHBG is produced primarily in hepatocytes, and it transports testosterone and other steroids in the blood plasma, reduces their metabolic clearance rate, and regulates the free testosterone concentration [34]. Therefore we speculated that the variation of synthesis function of SHBG may contribute to the disrupted reproductive hormone profiles.

PSA was produced by prostate luminal epithelial cells and regulated by androgen [35]. However, the association between circulating androgen and PSA levels is still unclear in general populations of men. Our study didn’t find any relationship between reproductive hormone levels and total PSA or free PSA except that SHBG was positively associated with free PSA (β = 0.003, *p* = 0.003), after multivariable adjustment. This is inconsistent with another study which indicated that men with lower TT or higher SHBG had lower total PSA [36]. Interestingly, we also found a positive association of LH and f/tPSA ratio (β = 0.002, *p* = 0.006). Xiong *et al* demonstrated that LH-LHR pathway played a critical role in the synthesis and secretion of PSA and other protein expressions in prostate cells [37]. Thus the positive association of LH and f/tPSA ratio may be due to the regulation of LH on androgen levels, and then affecting the secretion of PSA. Our study also showed negative relationships between TT, FT and I-PSS. This indicated that higher levels of TT and FT were protective factors for LUTS. This was inconsistent with another two studies which indicated no impact of endogenous TT on LUTS status [38,39]. Approximately 44% and 69% of participants in our previous study were more than 50 and 60 years old, respectively. It is commonly accepted that LUTS in older men are more prevalent than younger men [11]. With aging, a significant percentage of men develop late-onset hypogonadism (LOH), which is characterized by a deficiency in testosterone levels [40]. Taken together, higher level of TT and FT in older men may play a protective role for LUTS status in a complex way which is still unclear and need further investigation.

To date, this is the largest study in Chinese population to evaluate the effect of alcohol consumption on prostate measurements, and our study was based on a multi-centre community population. Furthermore, alcohol consumption in our study was characterized in detail, using a number of different measures. The measurements in this study were performed from the perspective of serum markers (total PSA, free PSA and f/tPSA ratio) and self-reported symptom (I-PSS). We realize that this study was not without limitations. First, we didn’t carry out a diagnosis of prostate diseases for each subject in this study, thus the association between alcohol consumption and specific prostate diseases incidence could not be analyzed. Second, we didn’t use a random sampling method for selection of participants, but we used the method of cluster sampling, the former one is more representative of the whole population in epidemiological study. Additional studies should be carried out to investigate the effect of alcohol consumption on the risk of specific prostate diseases in Chinese population, and to investigate the mechanisms by which alterations in reproductive hormones may mediate disruption changes of prostate measurements caused by alcohol consumption.

## Conclusions

In this cross-sectional study of drinking habits and markers of prostate health as well as reproductive hormone profiles in Chinese men, we observed that current drinkers had, on average, lower value of free PSA and f/tPSA ratio, former drinkers also had lower level of free PSA compared with never drinkers, whereas alcohol consumption had no obvious impact on prostate-related symptomatology as measured using the I-PSS. Alcohol consumption acts as an endocrine disruptor on the male reproductive hormone profiles, especially for LH, estridiol, FT and SHBG. Moreover, LH was found to be positively associated with f/tPSA ratio, SHBG was also positively related with free PSA and f/tPSA ratio, higher level of TT and FT may be protective for LUTS status. Further study should be carried out to reveal the the mechanisms by which disrupted hormone profiles may mediate alteration of prostate measurements caused by alcohol consumption.
